# SPAK Deficiency Attenuates Chemotherapy-Induced Intestinal Mucositis

**DOI:** 10.3389/fonc.2021.733555

**Published:** 2021-11-23

**Authors:** Tien-Yu Huang, Sung-Sen Yang, Ching-Len Liao, Ming-Hong Lin, Hsuan-Hwai Lin, Jung-Chun Lin, Peng-Jen Chen, Yu-Lueng Shih, Wei-Kuo Chang, Tsai-Yuan Hsieh

**Affiliations:** ^1^ Division of Gastroenterology, Department of Internal Medicine, Tri-Service General Hospital, National Defense Medical Center, Taipei, Taiwan; ^2^ Taiwan Association for the Study of Small Intestinal Diseases, Taoyuan, Taiwan; ^3^ Division of Nephrology, Department of Internal Medicine, Tri-Service General Hospital, National Defense Medical Center, Taipei, Taiwan; ^4^ Graduate Institute of Medical Sciences, National Defense Medical Center, Taipei, Taiwan; ^5^ Institute of BioMedical Science, Academia Sinica, Taipei, Taiwan; ^6^ Department of Microbiology and Immunology, National Defense Medical Center, Taipei, Taiwan; ^7^ Department of Microbiology and Immunology, College of Medicine, Kaohsiung Medical University, Kaohsiung, Taiwan

**Keywords:** chemotherapy-induced intestinal mucositis, gut homeostasis, enterocytes, tight junctions, small intestine, 5-fluorouracil

## Abstract

**Introduction:**

Ste20-related protein proline/alanine-rich kinase (SPAK) affects cell proliferation, differentiation, and transformation, and sodium and chloride transport in the gut. However, its role in gut injury pathogenesis is unclear.

**Objective:**

We determined the role of SPAK in chemotherapy-induced intestinal mucositis using *in vivo* and *in vitro* models.

**Methods:**

Using SPAK-knockout (KO) mice, we evaluated the severity of intestinal mucositis induced by 5-fluorouracil (5-FU) by assessing body weight loss, histological changes in the intestinal mucosa, length of villi in the small intestine, pro-inflammatory cytokine levels, proliferative indices, and apoptotic indices. We also evaluated changes in gut permeability and tight junction-associated protein expression. Changes in cell permeability, proliferation, and apoptosis were assessed in SPAK siRNA-transfected 5FU-treated IEC-6 cells.

**Results:**

5-FU-treated SPAK-KO mice exhibited milder intestinal mucositis, reduced pro-inflammatory cytokine expression, increased villus length, good maintenance of proliferative indices of villus cells, decreased apoptotic index of enterocytes, reduced gut permeability, and restoration of tight junction protein expression (*vs*. 5-FU-treated wild-type mice). Under *in vitro* conditions, siRNA-mediated SPAK-knockdown in IEC-6 cells decreased cell permeability and maintained homeostasis following 5-FU treatment.

**Conclusion:**

SPAK deficiency attenuated chemotherapy-induced intestinal mucositis by modulating gut permeability and tight junction-associated protein expression and maintaining gut homeostasis in murine small intestinal tissues following gut injury. The expression of SPAK may influence the pathogenesis of chemotherapy-induced intestinal mucositis.

## Introduction

Chemotherapy-induced intestinal mucositis is a major cause of morbidity and mortality in patients with cancer (1), resulting in severe diarrhea, malabsorption, infection, and reduced chemotherapeutic efficacy. The drug 5-fluorouracil (5-FU) is a common and effective adjuvant administered for gastrointestinal malignancies and improves disease-free survival and survival duration (1–3). However, marked diarrhea develops in approximately 50%–80% of patients who are administered 5-FU or other chemotherapeutic agents (3). Chemotherapy-induced intestinal mucositis is characterized by a decrease in the villus length, an increase in mucosal permeability, and disruption of crypt cell homeostasis (4–6). Mucositis results from direct chemotherapy-induced cytotoxicity and abnormal inflammatory processes (7). Currently, known strategies for attenuating chemotherapy-induced intestinal damage have been primarily ineffective (3), and intestinal tissue protection is typically neglected during chemotherapy (8).

Chemotherapy induces damage to both DNA and other cellular components, followed by a variety of inflammatory responses (7, 9). Chemotherapy also disrupts the epithelial layer and intestinal progenitor cells, subsequently increasing gut permeability. Such an increase in gut permeability is strongly associated with gut inflammation and injury (10, 11). In cancer patients, there is a strong relationship between gut permeability and the severity of chemotherapy-induced gut damage (6). In the injured gut, increased permeability triggers translocation of gut microbial products and the inflammatory process (12). The maintenance of homeostasis and the healing abilities of the gut mucosa also function to ameliorate gut injury.

In gut epithelial cells, Ste20-related proline/alanine-rich kinase (SPAK) is involved in the regulation of intestinal barrier function (13). SPAK also mediates pro-inflammatory cytokine expression and gut permeability in a colitis mouse model. SPAK interacts with inflammation-related kinases, the transcription factor AP-1, and the nuclear factor of kappa B (NF-κB) pathway, exerting diverse biological effects such as regulation of cell differentiation, proliferation, cytoskeleton rearrangement, and chloride transport (14–16). Therefore, SPAK protein could play an important role in regulating intestinal inflammation or injury (13, 15). Recent studies in transgenic mouse models have shown that overexpression of SPAK protein exacerbates the severity of murine colitis by increasing gut permeability (17). Moreover, Yan and colleagues reported that, in SPAK-knockout (KO) mice, SPAK deficiency could attenuate the severity of chemical-induced acute colitis by reducing epithelial permeability and increasing intestinal immune homeostasis (18). In addition, tight junction-associated proteins (e.g., JAM-A, occludins, and ZO-1) are strongly associated with gut permeability, and their expression is correlated with the severity of gut damage (19–21). However, the specific mechanisms underlying the regulation of mucosal permeability and tight junction proteins mediated by SPAK during gut damage are unclear. Our understanding of the roles of SPAK and the interactions between SPAK and permeability-associated molecules or pathways in the pathogenesis of chemotherapy-induced intestinal mucositis is limited.

Therefore, in this study, we used a SPAK-KO mouse model of 5-FU-induced intestinal mucositis to determine the association of SPAK protein with gut permeability. Additionally, we evaluated the role of SPAK *in vitro* using siRNA-mediated knockdown of SPAK in IEC-6 cells.

## Methods

### Animals

SPAK-KO and wild-type (WT, C57BL/6 strain) mice were bred and housed at the Laboratory Animal Center of the National Defense Medical Center (NDMC). All animal experiments were approved by the Institutional Animal Care and Use Committee of NDMC. All studies adhered to the Declaration of Helsinki and the institutional guidelines of the NDMC.

### 5-FU-Induced Intestinal Mucositis

Intestinal mucositis was induced in mice (SPAK-KO and WT mice, 6–8 weeks of age, male) *via* intraperitoneal injection of 5-FU (30 mg/kg/day; 10 ml/kg/per injection; Sigma Chemicals) on days 0–4 of the study duration. In our preliminary study, we compared the differences between male and female mice, and found that the clinical changes were similar. Therefore, we used male mice for further investigations. In the observation model, changes in body weight and stool consistency score (stool consistency scoring: “0” indicated normal stool, “1” indicated slight diarrhea [slightly wet and soft stool], “2” indicated moderate diarrhea [wet and unformed stool], and “3” indicated watery diarrhea) in mice were recorded daily from days 0 to 8, as described previously (22). Another animal model (using the same protocol) was established to assess histopathological changes in the small intestine in mice. Mice were sacrificed on day 5, which was the time of prominent gut injury in the 5-FU-induced intestinal mucositis model, as observed in our previous study (22).

### Assessment of the Severity of 5-FU-Induced Intestinal Mucositis

To assess the severity of the changes in the guts of mice with 5-FU-induced intestinal mucositis, the tissues of the proximal jejunum and distal ileum were dissected on day 5, fixed, embedded, stained with hematoxylin and eosin (H&E), and evaluated histologically. The lengths of the villi in the jejunum and ileum were measured microscopically.

### Cell Culture and Transfection

Murine intestinal epithelial IEC-6 cells (Bioresource Collection and Research Center, Hsinchu, Taiwan) were cultured in Dulbecco’s modified Eagle’s medium containing 5% fetal bovine serum, 1.5 g/L sodium bicarbonate, 4.5 g/L glucose, and 10 mM sodium pyruvate supplemented with 0.1 U/mL bovine insulin, as described previously (22). For siRNA transfection, siRNA targeting murine SPAK, and control siRNA were purchased from Dharmacon. SPAK siRNA-treated cells and control cells were cultured on glass coverslips for 18 h and then in fresh medium alone or with 5-FU.

### Permeability Assays Under *In Vivo* and *In Vitro* Conditions

Permeability assays were performed as described previously (23). Briefly, the fluorescein isothiocyanate (FITC)-labeled dextran method was used to assess barrier function. This method is an indirect approach for measuring total intestinal permeability. For the *in vivo* assay, on day 5, mice were denied access to water and food for 4 h. Subsequently, mice were administered FITC-labeled dextrans (60 mg/100 g body weight; Sigma-Aldrich, St. Louis, MO, USA) as a permeability tracer *via* gavage. Serum was collected retro-orbitally at 4 h after gavage. The fluorescence intensity of the serum was measured (485 nm excitation/520 nm emission; Cytofluor 2300; Waters Chromatography, Millipore, Billerica, MA, USA), and the FITC-dextran concentrations were determined by using standard curves generated by serial dilution of FITC-dextran. For *in vitro* permeability assays, confluent and polarized IEC-6 cells grown on transwell filters (membrane pores: 0.4 μm) in 12-well plates were treated with 5 and 10 μM 5-FU for 24 h. Then, the cells were further treated with FITC-labeled dextrans (4 kDa; Sigma-Aldrich) placed in the upper chamber for 2 h at 37°C. The medium in the lower chamber was collected and used for permeability assays. Based on the results of our pilot study, the dose-dependent test, and our previous study (22), we chose 10 uM 5-FU as the optimal concentration for the *in vitro* study.

### Determination of the Expression of Pro-Inflammatory and Tight Junction-Associated Genes in Small Intestinal Tissues Using Real-Time Reverse Transcription-Polymerase Chain Reaction (RT-PCR)

On day 5, the small intestine was removed and homogenized. Total RNA was extracted using an RNeasy Mini Kit (Qiagen, Valencia, CA, USA). Real-time RT-PCR products were analyzed using an iCycler iQ instrument (Bio-Rad Laboratories, Hercules, CA, USA) with the primers listed in **Supplementary Table 1**.

### Immunohistochemical Staining of the Sections of the Small Intestine

Immunohistochemical staining of the small intestine was performed using LSAB2 HRP kits (DakoCytomation). Primary antibodies against tumor necrosis factor (TNF)-α, SPAK, JAM-A, claudin, occludin, and ZO-1 were used for the immunohistochemical analysis.

### Terminal Deoxynucleotidyl Transferase dUTP Nick-End Labeling (TUNEL) Assay of Small Intestine Sections

Enterocyte apoptosis in the small intestine was detected using TUNEL assays with an *in situ* apoptosis detection kit (S7101; Chemicon Inc.), according to the manufacturer’s instructions.

### Detection of Proliferating Enterocytes in the Small Intestine

Monoclonal antibodies (Santa Cruz Biotechnology, Santa Cruz, CA, USA) specific for proliferating cell nuclear antigen (PCNA) were used for the immunohistochemical analysis of the proliferation capability of crypt cells.

### Cell Cytometry

Control IEC-6 cells and those with SPAK-knockdown (KD) were cultured for 3 h in the presence of 10 μM 5-FU or dimethyl sulfoxide (DMSO). For PCNA staining, cells were harvested and fixed in fixation buffer for 20 min at room temperature. Fixed cells were then stained with FITC-conjugated anti-rat PCNA antibodies (clone PC10; eBioscience) for 30 min at room temperature. For the analysis of cell apoptosis, solvent- or 5-FU-treated fresh cells were stained with FITC-conjugated annexin-V (eBioscience) for 30 min on ice. All stained cells were then analyzed using a FACSCalibur flow cytometry instrument (BD Pharmingen, San Jose, CA, USA), and data were processed using the FlowJo software (Tree Star, Ashland, OR, USA).

### Statistical Analysis

All data were analyzed using GraphPad Prism 4 and are presented as the mean ± standard error of the mean (SEM). Differences between groups were assessed using analysis of variance (ANOVA) and nonparametric Mann-Whitney U tests. Survival rates were evaluated using the Kaplan-Meier procedure and log-rank tests.

## Results

### SPAK Deficiency Attenuated the Severity of Intestinal Mucositis Induced by 5-FU in Mice

**Figure 1:**
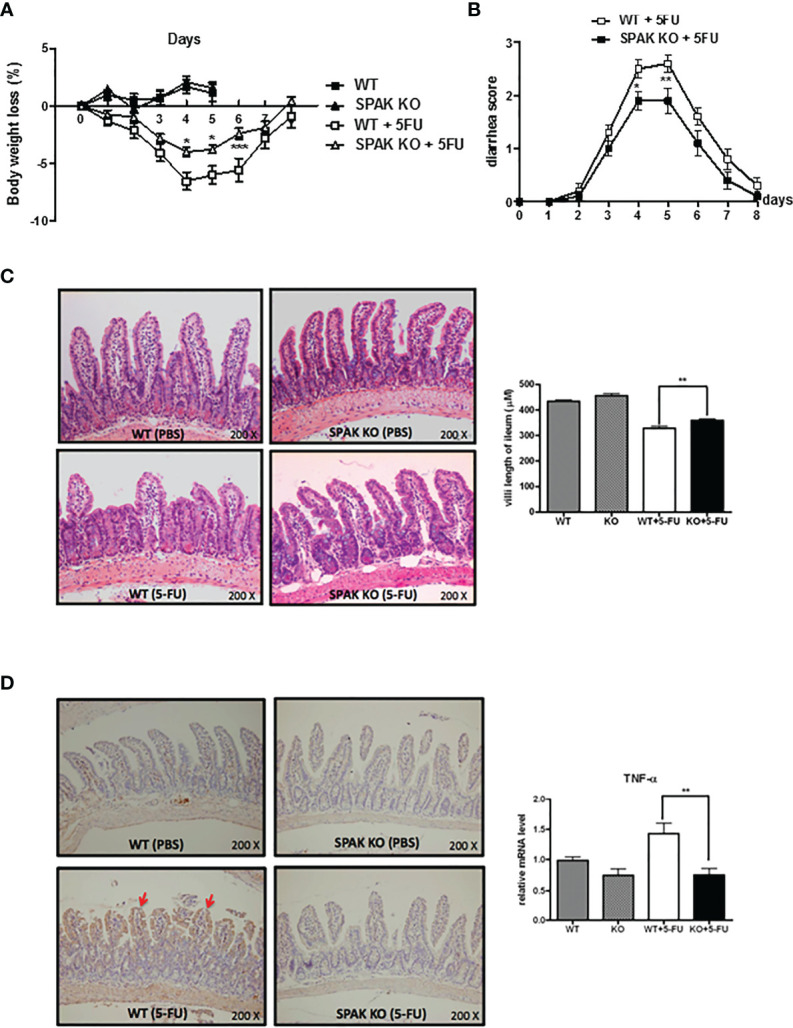
Ste20-related protein proline/alanine-rich kinase (SPAK) deficiency attenuated the severity of 5-fluorouracil (FU)-induced intestinal mucositis and suppressed the expression of pro-inflammatory cytokines in the small intestines of 5-FU-treated mice. **(A)** SPAK-knockout (KO) and wild-type (WT) mice treated with 5-FU were monitored daily for a reduction in body weight (n = 5–10/group). **(B)** SPAK-knockout (KO) and wild-type (WT) mice treated with 5-FU were monitored daily for diarrhea status (n =10/group). **(C)** Representative hematoxylin and eosin (H&E)-stained histological sections of the ilea are shown (original magnification: 200×). Some mice in each group (n = 5) were euthanized on day 5 to determine the effects of the treatment on the lengths of villi in the ilea. Microscopic measurements of 20–30 villi at 100× magnification were performed using ileal sections obtained from each mouse. **(D)** Representative immunohistochemical staining of tumor necrosis factor (TNF)-α on ileal sections is shown (the red arrows indicate the areas that were positive for TNF-α, original magnification: 200×). Relative mRNA levels of *TNF-α* in the small intestines of WT mice and SPAK-KO mice treated with 5-FU (n = 5–10/group). Each point or bar represents the mean ± standard error of the mean (SEM). *P < 0.05, **P < 0.01, and ***P < 0.001 indicate differences between groups of WT mice that received 5-FU and SPAK mice treated with 5-FU.

In WT mice with 5-FU-induced intestinal mucositis, which resembles human 5-FU-induced intestinal mucositis, we observed progressive loss of body weight and diarrhea, consistent with our previous report (22). Bodyweight was regained gradually after discontinuing the administration of 5-FU on day 6. However, compared with that in WT mice, SPAK-KO mice exhibited significantly reduced loss of body weight on days 4–6 (**Figure 1A**) and less severe diarrhea (**Figure 1B**). Microscopic examination of the integrity and architecture of the small intestines, showed that mice with 5-FU-induced intestinal mucositis had markedly decreased villus length and crypt depth (data not shown) in the proximal jejunum and distal ileum, compared with phosphate-buffered saline-treated WT controls (**Figure 1C** and **Supplementary Figure 1**). In contrast, SPAK deficiency restored the length of the villi and preserved the architecture of the intestinal mucosa in mice with 5-FU-induced intestinal mucositis compared with those in WT mice (**Figure 1C** and **Supplementary Figure 1**).

### SPAK Deficiency Inhibited the Expression of Pro-Inflammatory Cytokines in the Small Intestines of Mice With 5-FU-Induced Intestinal Mucositis

In the pathogenesis of chemotherapy-induced intestinal mucositis, induction and amplification of the expression of components of inflammatory pathways and pro-inflammatory cytokines, such as IL-1β and TNF-α, play important roles. Therefore, we examined the mRNA and protein expression of IL-1β and TNF-α in the small intestinal tissues of mice following the 5-FU treatment. Immunohistochemical staining showed that the expression of TNF-α protein increased in the small intestinal mucosa of 5-FU-treated mice (**Figure 1D**). In addition, *IL-1β* and *TNF-α* mRNA levels were upregulated in the small intestines of WT mice treated with 5-FU (**Figure 1D** and **Supplementary Figure 2**). In contrast, SPAK deficiency inhibited the upregulation of IL-1β and TNF-α in the small intestines of mice treated with 5-FU (**Figure 1D** and **Supplementary Figure 2**).

### SPAK Deficiency Mitigated the Increased Gut Permeability in Mice Treated With 5-FU

**Figure 2:**
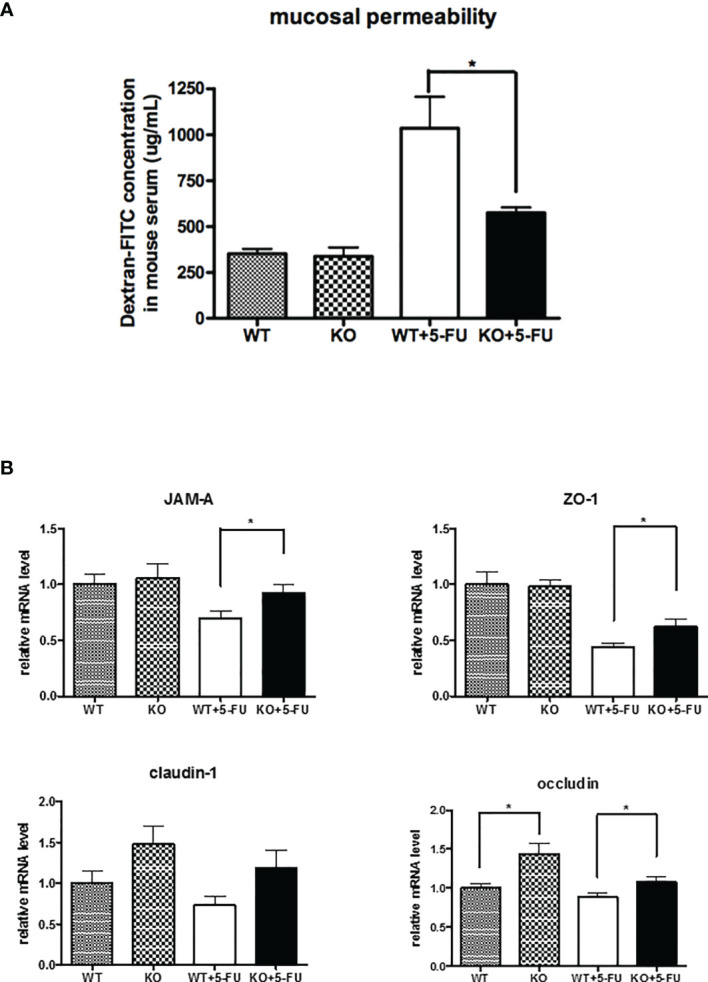
SPAK deficiency attenuated the increased gut permeability and restored the downregulation of tight junction-associated proteins in the small intestines of mice with 5-FU-induced intestinal mucositis. **(A)** Gut permeability of WT and SPAK mice treated with 5-FU was determined by fluorescein isothiocyanate (FITC)-labeled dextran assays (WT: n = 5, KO: n = 5, WT+5-FU: n = 6, KO+5-FU: n = 6). **(B)** Relative mRNA levels of tight junction-associated proteins in the small intestines of WT mice and SPAK-KO mice treated with 5-FU (WT: n = 9, KO: n = 9, WT+5-FU: n = 10, KO+5-FU: n = 10). Each point or bar represents the mean ± SEM; *P < 0.05 indicates differences between groups of WT mice that received 5-FU and SPAK mice treated with 5-FU.

We determined the effects of SPAK protein on changes in gut permeability of mice treated with 5-FU. Assays examining FITC-labeled dextran showed that 5-FU treatment markedly increased gut permeability in mice (**Figure 2A**). However, SPAK deficiency attenuated the increased gut permeability in mice with 5-FU-induced intestinal mucositis (**Figure 2A**).

### SPAK Deficiency Restored the Downregulation of the mRNA Levels of Tight Junction-Associated Proteins in the Small Intestines of Mice With 5-FU-Induced Intestinal Mucositis

As gut permeability is closely associated with the expression and location of tight junction-associated proteins, we further examined the mRNA expression of tight junction-associated proteins in the small intestines of mice. The expression of occludin mRNA in the small intestines of SPAK-KO control mice was significantly higher than that in WT control mice (**Figure 2B**). A trend of elevated mRNA expression of claudin-1 in the small intestines of SPAK-KO mice was also noted. However, no significant differences in *JAM-A* or *ZO-1* expression were observed. Following 5-FU treatment, the expression of mRNAs of tight junction-associated proteins, including JAM-A, ZO-1, and occludin was downregulated in the small intestines of WT mice (**Figure 2B**). However, SPAK-KO mice treated with 5-FU exhibited higher levels of mRNAs encoding tight junction-associated proteins in the small intestines than 5-FU-treated WT mice (**Figure 2B**).

### SPAK Deficiency Restored the Proliferation of Crypt Cells in the Small Intestines in Response to 5-FU Chemotherapy

**Figure 3:**
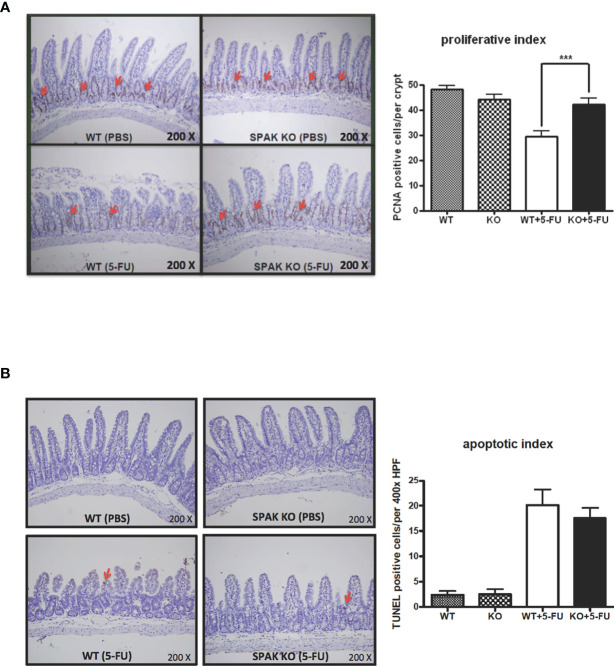
Effects of SPAK on the proliferation and apoptosis of enterocytes in the small intestine of WT and SPAK-KO mice with 5-FU-induced intestinal mucositis. **(A)** Representative immunohistochemical staining of proliferating cell nuclear antigen (PCNA) in sections of the ileum is shown (the red arrows indicate positive cells, original magnification: 200×). The proliferation index was defined as the average number of PCNA-positive cells per crypt in 6–9 different microscopic fields observed at 400× magnification in sections of the ileum (n = 3/group). **(B)** Representative terminal deoxynucleotidyl transferase dUTP nick-end labeling (TUNEL) assays of ileum sections are shown (the red arrows indicate positive cells, original magnification: 200×). The apoptotic index was defined as the average number of TUNEL-positive enterocytes per field in 5–6 continuous microscopic fields observed at 400× magnification in sections of distal ileal tissue from mice sacrificed on day 5 after 5-FU treatment (n = 3/group). Each bar represents the mean ± SEM; ***P < 0.001 represents significant differences between the indicated pairs.

Gut homeostasis is associated with the pathogenesis of gut inflammation or injury. Chemotherapy markedly impairs the homeostasis of the gut mucosa. Therefore, we next evaluated proliferation and apoptosis in the small intestines in response to 5-FU chemotherapy. 5-FU chemotherapy could increase the apoptotic index and decrease the proliferation index in the small intestines of mice (**Figures 3A, B**), consistent with our previous report (22). Interestingly, SPAK deficiency restored the proliferation of crypt cells in the small intestines of mice treated with 5-FU (**Figure 3A**). In addition, SPAK deficiency showed a tendency to decrease the apoptotic index of the mucosa in the small intestines of mice with 5-FU-induced intestinal mucositis; however, no statistically significant difference was observed (**Figure 3B**).

### Knockdown of SPAK in IEC-6 Cells Decreased Cell Permeability Following Treatment With 5-FU

**Figure 4:**
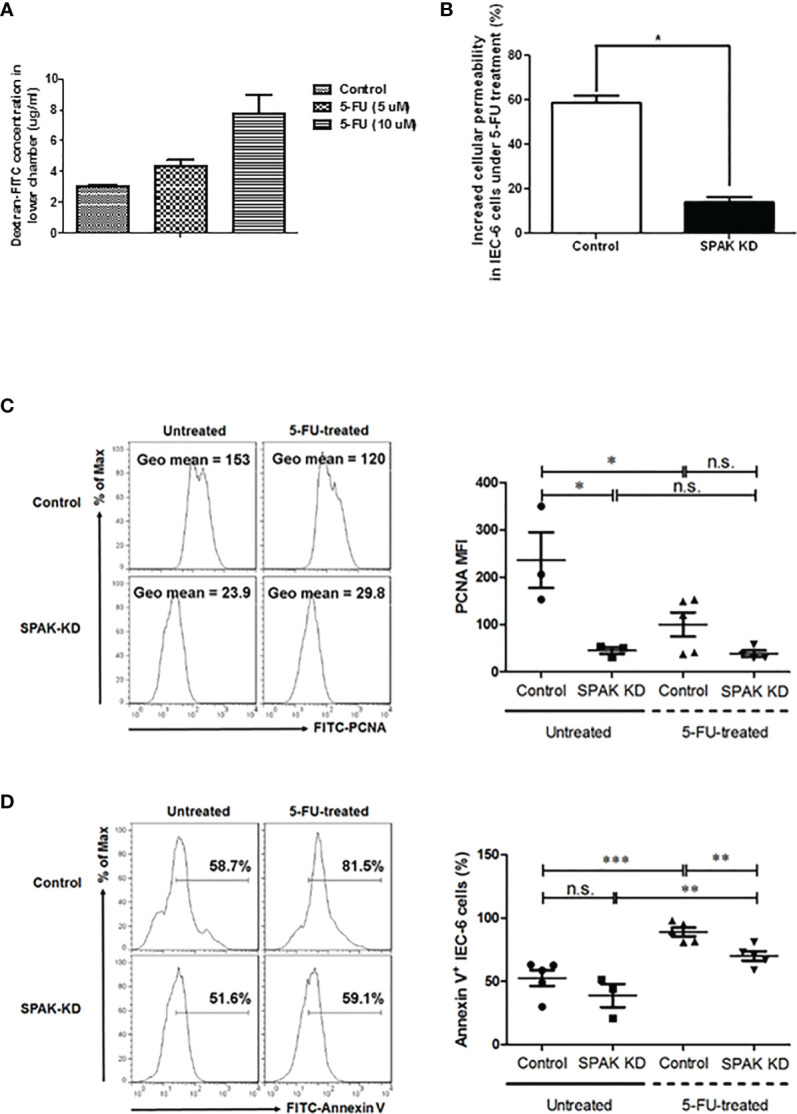
**(A, B)** Cell permeability in murine epithelial IEC-6 cells following treatment of 5-FU. **(A)** After 5-FU treatment, permeability of IEC-6 cells increases in a concentration-dependent manner (5 and 10 μM). **(B)** siRNA-mediated knockdown of *SPAK* expression in IEC-6 cells blocks this increase in cell permeability compared to control cells treated with 5-FU (10 μM). **(C, D)** SPAK-knockdown modulates the effects of 5-FU treatment on the proliferation and survival of IEC-6 cells. Control and SPAK-KD IEC-6 cells were treated with 10 µM 5-FU and dimethyl sulfoxide (DMSO) solvent for 3 h, respectively. Untreated and 5-FU-treated control or SPAK-KD IEC-6 cells were analyzed to determine the mean fluorescence index (MFI) of PCNA expression **(C)** and the percentage of annexin-V-positive cells **(D)**, respectively. **(C)** Representative flow cytometry assays are shown in the left panel, and the MFI of PCNA is shown in the right panel. **(D)** Representative flow cytometry assays (left panel) and the frequencies (right panel) of annexin-V-positive IEC-6 cells are presented. Data corresponding to MFIs or frequencies are representatives of at least three mice in each group, presented as the mean ± SEM. Two-tailed Student’s unpaired *t*-tests were used for statistical analysis. ***P < 0.0001; **P < 0.01; *P < 0.05 n.s., not significant.

To further elucidate the role of SPAK in gut permeability, we evaluated permeability of murine epithelial IEC-6 cells following transfection with SPAK siRNA. After 5-FU treatment, IEC-6 cell permeability increased in a concentration-dependent manner (**Figure 4A**). In contrast, siRNA-mediated knockdown of *SPAK* expression in IEC-6 cells blocked this increase in cell permeability compared to control cells treated with 5-FU (**Figure 4B**). To further confirm whether SPAK downregulation affected the proliferative ability of murine intestinal epithelial cells following 5-FU treatment, we analyzed the expression of PCNA in SPAK-KD IEC-6 cells after 5-FU treatment for 3 h. Consistent with our previous results, the intensity of PCNA expression was significantly lower in 5-FU-treated IEC-6 cells than in untreated control cells (**Figure 4C**), suggesting a suppressive role of 5-FU treatment in the proliferation of intestinal epithelial cells. Nevertheless, the proliferation of SPAK-KD IEC-6 cells was not suppressed after 5-FU treatment. These results suggested that SPAK downregulation at least restored the suppression of 5-FU-treated IEC-6 cell proliferation. Moreover, to further confirm whether SPAK modulated the apoptosis of intestinal epithelial cells following 5-FU treatment, we examined the average levels of annexin-V in SPAK-KD IEC-6 cells treated with 5-FU. Treatment with 5-FU markedly increased apoptosis in IEC-6 cells in comparison with untreated control cells (**Figure 4D**), consistent with our previous results (22). In contrast, the expression level of annexin-V was significantly lower in 5-FU-treated SPAK-KD IEC-6 cells than that in control cells. Nevertheless, SPAK-KD did not affect the survival of untreated IEC-6 cells (**Figure 4D**). These results suggested that SPAK downregulation markedly rescued the suppression of apoptosis in 5-FU-treated IEC-6 cells.

## Discussion

**Figure 5:**
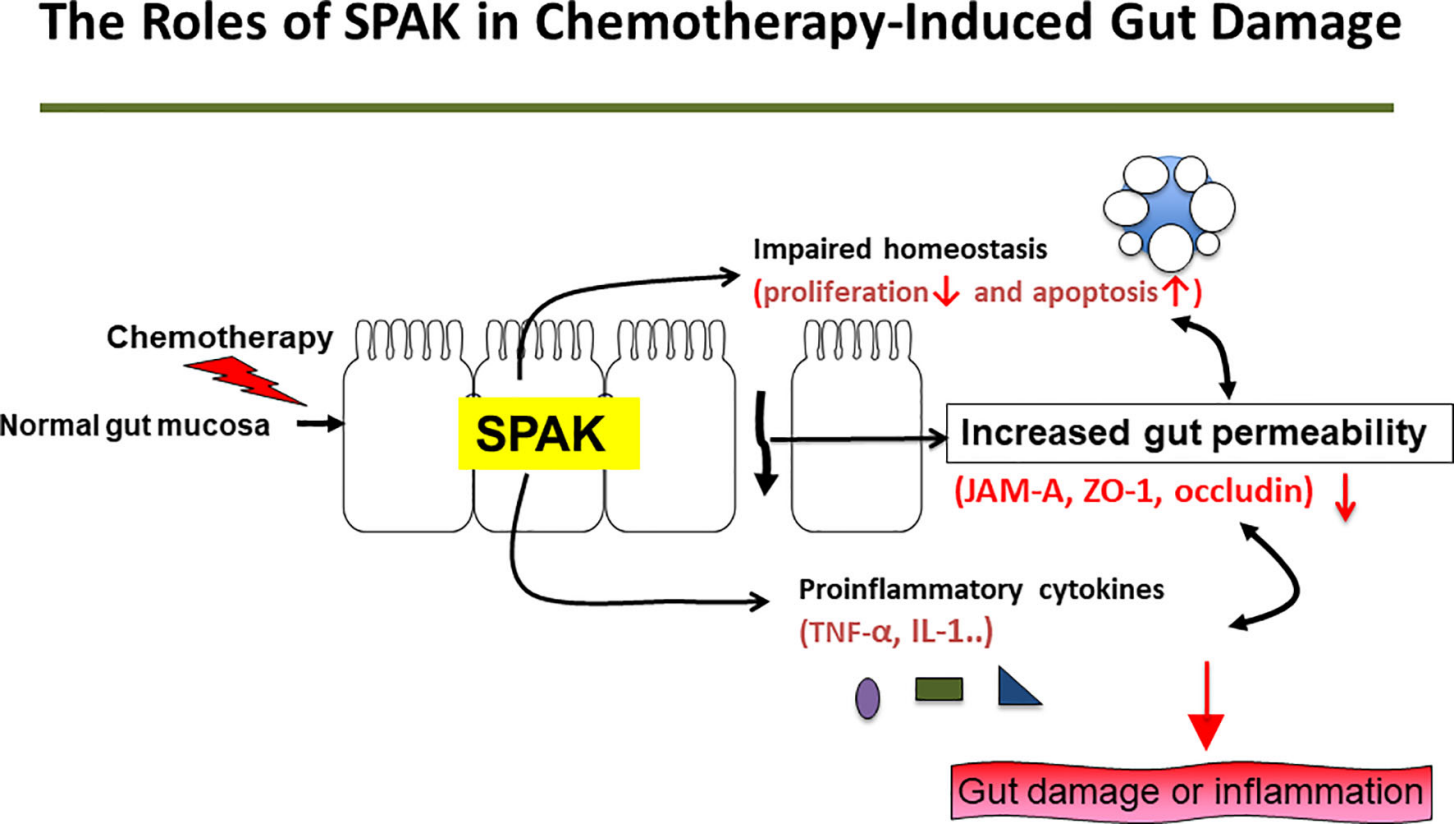
Potential mechanisms underlying effects of SPAK in the pathogenesis of 5-FU-induced intestinal mucositis.

SPAK is associated with cell differentiation, transformation, and chloride transport, in addition to a variety of other biological roles. SPAK protein is associated with chronic gut inflammation *via* regulation of gut permeability (13) and plays an important role in chemical-induced colitis (17, 18). However, the mechanisms involved in these processes are still unclear. In this study, we demonstrated for the first time, that SPAK deficiency could attenuate the severity of chemotherapy-induced intestinal mucositis *via* modulation of gut permeability and homeostasis *in vivo* and *in vitro*. Thus, our data provide important insights into the mechanisms underlying the regulation of gut permeability and homeostasis by SPAK (**Figure 5**).

In the pathogenesis of chemotherapy-related intestinal mucositis, initial DNA damage and subsequent inflammatory processes play important roles. In addition, disruption of the epithelial barrier affecting gut permeability and the function of tight junction could translocate the bacteria and bacterial products into the systemic circulation, resulting in sepsis. Subsequent induction and amplification of the expression of pro-inflammatory cytokines and inflammatory pathways contribute to mucosal damage in chemotherapy-induced intestinal mucositis (7). Pro-inflammatory cytokines such as IL-1β and TNF-α play a role in amplifying mucosal damage (7, 24). In this study, SPAK deficiency decreased the overexpression of *IL-1β* and *TNF-α* mRNA in small intestinal tissues of mice treated with 5-FU. SPAK also triggers the activation of the MAPK cascade and induces biological responses and inflammatory cascades, including indirect induction of IL-1 and TNF-α expression. However, downregulation of mRNA expression of *IL-1β* and *TNF-α* in SPAK-deficient mice and cells may be caused by both direct and indirect effects. The detailed mechanisms underlying these changes need to be determined further.

In the pathogenesis of chemotherapy-induced intestinal mucositis, intestinal homeostasis is disrupted. Inhibiting enterocyte proliferation and increasing enterocyte apoptosis could induce gut damage following chemotherapy. Therefore, maintaining gut or epithelial homeostasis (increased proliferation or inhibition of apoptosis during chemotherapy) could attenuate gut inflammation or damage (7, 22, 25). SPAK is involved in mediating apoptosis *via* the p38 or SPAK/JNK pathways (13). However, the role of SPAK protein in the pathogenesis of chemotherapy-induced intestinal mucositis is still unknown. In our study, SPAK deficiency restored the proliferation of enterocytes and tended to inhibit apoptosis in enterocytes in the small intestine following 5-FU treatment. Moreover, under *in vitro* conditions, siRNA-mediated knockdown of SPAK in ICE-6 cells restored cell proliferation and inhibited apoptosis following 5-FU treatment. These results revealed that SPAK could affect the homeostasis of epithelial cells in the small intestine of mice directly following 5-FU treatment. To the best of our knowledge, this is the first study showing the role of SPAK in gut homeostasis in chemotherapy-induced gut injury. However, the roles of SPAK in homeostasis in 5-FU-induced intestinal mucositis based on direct or indirect effects require further investigations.

Tight junction proteins are associated with gut permeability. JAM-KO mice exhibit increased mucosal permeability and altered expression of claudin and inflammatory cytokines in the colonic mucosa (19). SPAK-KO and transgenic mice exhibit altered gut permeability and tight junction protein expression (17, 18). In our study, we also demonstrated that SPAK deficiency could decrease epithelial permeability *in vivo* and *in vitro*. In addition, SPAK deficiency could restore the expression of JAM-A, ZO-1, and occludin. However, further studies are needed to examine the specific molecular mechanisms involved in these processes. Interestingly, SPAK deficiency increases the expression of occludin mRNA in the small intestinal tissues of untreated mice. Occlusions are important transmembranous and tight junction-associated proteins that influence gut permeability and mucosal homeostasis. Occludins may play a role in the effect of SPAK in the pathogenesis of 5-FU-induced intestinal mucositis.

Our study had some limitations. First, SPAK-KO strain mice exhibited less gut injury following 5-FU-induced mucositis, possibly attributable to improved permeability and mucosal homeostasis, as well as inhibition of pro-inflammatory cytokines under 5-FU treatment. Although the actual underlying mechanisms could not be clarified, 5-FU-related mucositis prevention through SPAK blocking was evident in our results. Comprehensive insights into the underlying mechanisms require further investigations. Second, we only used SPAK-KO and WT (C57BL/6 strain) mice for the study. As the tumor-bearing animal model is more similar to the actual human situation, it would provide more relevant information that could facilitate translational research. Third, there was no further analysis of the time-course of the study, which could provide more information regarding the role of SPAK in the pathogenesis of chemotherapy-induced mucositis.

In summary, chemotherapy-induced intestinal mucositis is a critical clinical issue that influences the quality of life of patients and treatment outcomes; prevention and control of this complication are important for both patients and physicians. In this study, we showed that SPAK might play an important role in the pathogenesis of chemotherapy-induced intestinal mucositis. SPAK was found to modulate gut permeability and homeostasis in the small intestine in 5-FU-treated mice and murine intestinal epithelial cells. Thus, SPAK could be a potential target for the treatment of chemotherapy-induced intestinal mucositis.

## Data Availability Statement

The original contributions presented in the study are included in the article/**Supplementary Material**. Further inquiries can be directed to the corresponding author.

## Ethics Statement

The animal study was reviewed and approved by the Institutional Animal Care and Use Committee of National Defense Medical Center, Taipei, Taiwan.

## Author Contributions

T-YHuang performed the experiments, analyzed the results, created the figures, and wrote the manuscript. M-HL analyzed the results of the flow cytometry and wrote the manuscript. H-HL, J-CL, P-JC, Y-LS, W-KC, and T-YHsieh edited the manuscript. T-YHuang, C-LL, and S-SY designed the experiments. All authors contributed to the article and approved the submitted version.

## Funding

Funding for this research was provided by the Tri-Service General Hospital (TSGH-C104-063, TSGH-C105-108), the Department of Defense (DOD-101-45), and the National Science Council (NSC 99-2314-B-016-041-MY2).

## Conflict of Interest

The authors declare that the research was conducted in the absence of any commercial or financial relationships that could be construed as a potential conflict of interest.

## Publisher’s Note

All claims expressed in this article are solely those of the authors and do not necessarily represent those of their affiliated organizations, or those of the publisher, the editors and the reviewers. Any product that may be evaluated in this article, or claim that may be made by its manufacturer, is not guaranteed or endorsed by the publisher.

## References

[1] McCarthy GM Awde JD Ghandi H Vincent M Kocha WI. Risk Factors Associated With Mucositis in Cancer Patients Receiving 5-Fluorouracil. Oral Oncol (1998) 34:484–90. doi: 10.1016/s1368-8375(98)00068-2 9930359

[2] Efficacy of Adjuvant Fluorouracil and Folinic Acid in Colon Cancer. International Multicentre Pooled Analysis of Colon Cancer Trials (IMPACT) Investigators. Lancet (1995) 345:939–44. doi: 10.1016/S0140-6736(95)90696-7 7715291

[3] Benson AB 3rd Ajani JA Catalano RB Engelking C Kornblau SM Martenson JA Jr. Recommended Guidelines for the Treatment of Cancer Treatment-Induced Diarrhea. J Clin Oncol (2004) 22:2918–26. doi: 10.1200/JCO.2004.04.132 15254061

[4] Cool JC Dyer JL Xian CJ Butler RN Geier MS Howarth GS. Pre-Treatment With Insulin-Like Growth Factor-I Partially Ameliorates 5-Fluorouracil-Induced Intestinal Mucositis in Rats. Growth Horm IGF Res (2005) 15:72–82. doi: 10.1016/j.ghir.2004.12.002 15701575

[5] Pritchard DM Potten CS Hickman JA. The Relationships Between P53-Dependent Apoptosis, Inhibition of Proliferation, and 5-Fluorouracil-Induced Histopathology in Murine Intestinal Epithelia. Cancer Res (1998) 58:5453–65. 9850079

[6] Melichar B Zezulová M. The Significance of Altered Gastrointestinal Permeability in Cancer Patients. Curr Opin Support Palliat Care (2011) 5:47–54. doi: 10.1097/SPC.0b013e328343a043 21326003

[7] Sonis ST. The Pathobiology of Mucositis. Nat Rev Cancer (2004) 4:277–84. doi: 10.1038/nrc1318 15057287

[8] Cascinu S Barni S Labianca R Del Ferro E Rocchi MB Ligi M. Evaluation of Factors Influencing 5-Fluorouracil-Induced Diarrhea in Colorectal Cancer Patients. An Italian Group for the Study of Digestive Tract Cancer (GISCAD) Study. Support Care Cancer (1997) 5:314–7. doi: 10.1007/s005200050079 9257428

[9] Leitão RF Ribeiro RA Bellaguarda EA Macedo FD Silva LR Oriá RB. Role of Nitric Oxide on Pathogenesis of 5-Fluorouracil Induced Experimental Oral Mucositis in Hamster. Cancer Chemother Pharmacol (2007) 59:603–12. doi: 10.1007/s00280-006-0301-y 16944152

[10] Schmitz H Barmeyer C Fromm M Runkel N Foss HD Bentzel CJ. Altered Tight Junction Structure Contributes to the Impaired Epithelial Barrier Function in Ulcerative Colitis. Gastroenterology (1999) 116:301–9. doi: 10.1016/s0016-5085(99)70126-5 9922310

[11] Berkes J Viswanathan VK Savkovic SD Hecht G. Intestinal Epithelial Responses to Enteric Pathogens: Effects on the Tight Junction Barrier, Ion Transport, and Inflammation. Gut (2003) 52:439–51. doi: 10.1136/gut.52.3.439 PMC1773546 12584232

[12] Sandler NG Koh C Roque A Eccleston JL Siegel RB Demino M. Host Response to Translocated Microbial Products Predicts Outcomes of Patients With HBV or HCV Infection. Gastroenterology (2011) 141:1220–30, 1230.e1–3. doi: 10.1053/j.gastro.2011.06.063 PMC3186837 21726511

[13] Yan Y Merlin D. Ste20-Related Proline/Alanine-Rich Kinase: A Novel Regulator of Intestinal Inflammation. World J Gastroenterol (2008) 14:6115–21. doi: 10.3748/wjg.14.6115 PMC2761571 18985800

[14] Yan Y Dalmasso G Nguyen HT Obertone TS Charrier-Hisamuddin L Sitaraman SV. Nuclear factor-kappaB Is a Critical Mediator of Ste20-Like Proline-/Alanine-Rich Kinase Regulation in Intestinal Inflammation. Am J Pathol (2008) 173:1013–28. doi: 10.2353/ajpath.2008.080339 PMC2543070 18787102

[15] Yan Y Nguyen H Dalmasso G Sitaraman SV Merlin D. Cloning and Characterization of a New Intestinal Inflammation-Associated Colonic Epithelial Ste20-Related Protein Kinase Isoform. Biochim Biophys Acta (2007) 1769:106–16. doi: 10.1016/j.bbaexp.2007.01.003 PMC1865517 17321610

[16] Polek TC Talpaz M Spivak-Kroizman T. The TNF Receptor, RELT, Binds SPAK and Uses it to Mediate P38 and JNK Activation. Biochem Biophys Res Commun (2006) 343:125–34. doi: 10.1016/j.bbrc.2006.02.125 16530727

[17] Yan Y Laroui H Ingersoll SA Ayyadurai S Charania M Yang S. Overexpression of Ste20-Related Proline/Alanine-Rich Kinase Exacerbates Experimental Colitis in Mice. J Immunol (2011) 187:1496–505. doi: 10.4049/jimmunol.1002910 PMC3140558 21705622

[18] Zhang Y Viennois E Xiao B Baker MT Yang S Okoro I. Knockout of Ste20-Like Proline/Alanine-Rich Kinase (SPAK) Attenuates Intestinal Inflammation in Mice. Am J Pathol (2013) 182:1617–28. doi: 10.1016/j.ajpath.2013.01.028 23499375

[19] Laukoetter MG Nava P Lee WY Severson EA Capaldo CT Babbin BA. JAM-A Regulates Permeability and Inflammation in the Intestine *In Vivo* . J Exp Med (2007) 204:3067–76. doi: 10.1084/jem.20071416 PMC2150975 18039951

[20] Vetrano S Ploplis VA Sala E Sandoval-Cooper M Donahue DL Correale C. Unexpected Role of Anticoagulant Protein C in Controlling Epithelial Barrier Integrity and Intestinal Inflammation. Proc Natl Acad Sci USA (2011) 108:19830–5. doi: 10.1073/pnas.1107140108 PMC3241765 22109555

[21] Zakostelska Z Kverka M Klimesova K Rossmann P Mrazek J Kopecny J. Lysate of Probiotic *Lactobacillus Casei* DN-114 001 Ameliorates Colitis by Strengthening the Gut Barrier Function and Changing the Gut Microenvironment. PLoS One (2011) 6:e27961. doi: 10.1371/journal.pone.0027961 22132181 PMC3222668

[22] Huang TY Chu HC Lin YL Ho WH Hou HS Chao YC. Minocycline Attenuates 5-Fluorouracil-Induced Small Intestinal Mucositis in Mouse Model. Biochem Biophys Res Commun (2009) 389:634–9. doi: 10.1016/j.bbrc.2009.09.041 19765544

[23] Yan Y Dalmasso G Nguyen HT Obertone TS Sitaraman SV Merlin D. Ste20-Related Proline/Alanine-Rich Kinase (SPAK) Regulated Transcriptionally by Hyperosmolarity Is Involved in Intestinal Barrier Function. PLoS One (2009) 4:e5049. doi: 10.1371/journal.pone.0005049 19343169 PMC2660421

[24] Korzenik JR Podolsky DK. Evolving Knowledge and Therapy of Inflammatory Bowel Disease. Nat Rev Drug Discov (2006) 5:197–209. doi: 10.1038/nrd1986 16518373

[25] Kurita A Kado S Kaneda N Onoue M Hashimoto S Yokokura T. Modified Irinotecan Hydrochloride (CPT-11) Administration Schedule Improves Induction of Delayed-Onset Diarrhea in Rats. Cancer Chemother Pharmacol (2000) 46:211–20. doi: 10.1007/s002800000151 11021738

