# Supplementary material for: SPAK Deficiency Attenuates Chemotherapy-Induced Intestinal Mucositis
Source: Front Oncol. 2021 Nov 23;11:733555. doi: 10.3389/fonc.2021.733555 (PMC8649624; doi:10.3389/fonc.2021.733555)
Supplement: Supplementary file 1 [file DataSheet_1.docx]

Supplementary Material

# Supplementary Figures and Tables

## Supplementary Figures


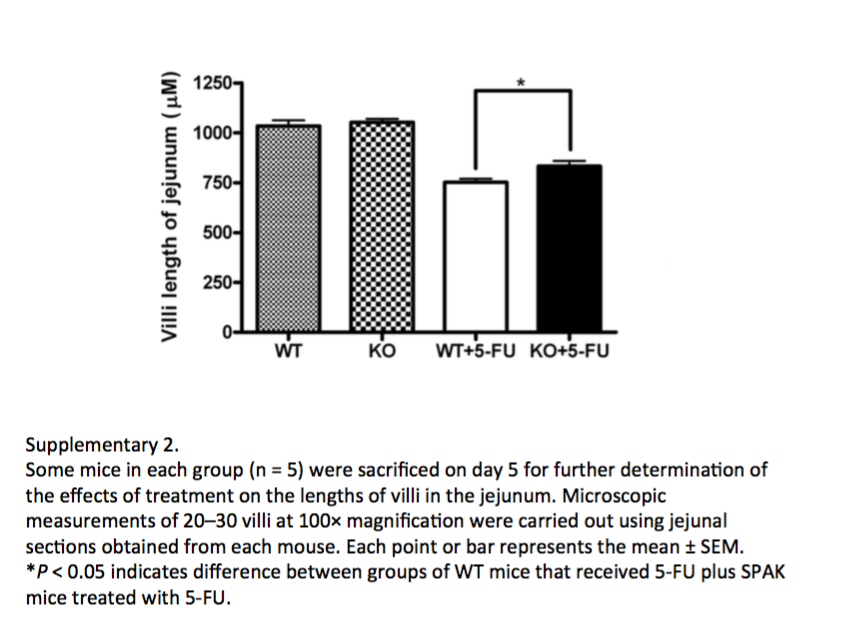


**Supplementary Figure 1.**


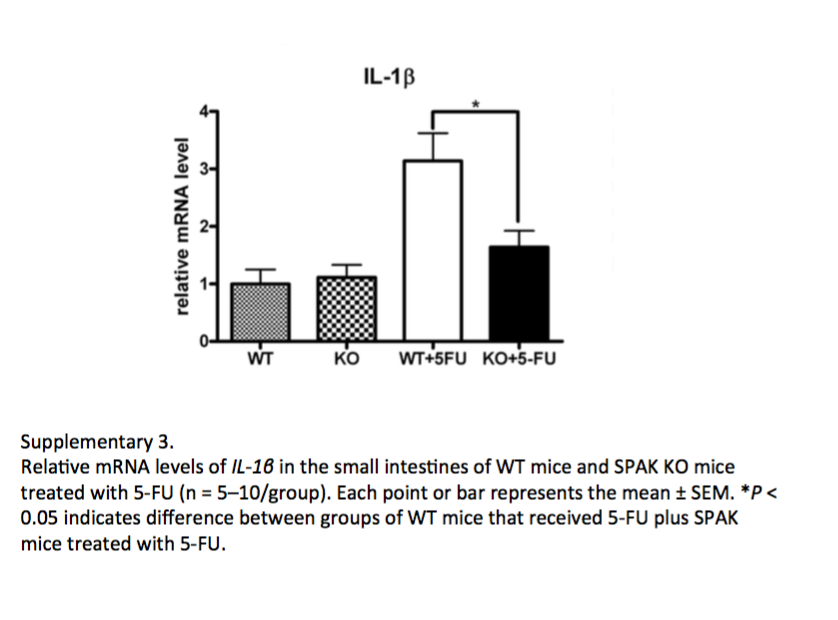


**Supplementary Figure 2.**

## Supplementary Tables

| Supplementary Table 1. Primers for quantitative RT-PCR | | |
| --- | --- | --- |
| Primer | | Sequence |
| *IL-1β* | Forward | 5′-TGAGCACCTTCTTTTCCTTCA-3′ |
|  | Reverse | 5′-GCAGCTGTCTAATGGGAACG-3′ |
| *TNF-α* | Forward | 5′-CTGTAGCCCACGTCGTAGC-3′ |
|  | Reverse | 5′-TTGAGATCCATGCCGTTG-3′ |
| *SPAK* | Forward | 5′-GTACGAGCTCCAGGAGGTTATC-3′ |
|  | Reverse | 5′-TCTTGCCTGGGTTTGCAT-3′ |
| *JAM-A* | Forward | 5′-agaacaaagaaagggactgcac-3′ |
|  | Reverse | 5′-accaggaacgacgaggtct-3′ |
| *Claudin-1* | Forward | 5′-cttgacccccatcaatgc-3′ |
|  | Reverse | 5′-cacctcccagaaggcaga-3′ |
| *Occludin* | Forward | 5′-tccgtgaggccttttgaa-3′ |
|  | Reverse | 5′-ggtgcataatgattgggtttg-3′ |
| *ZO-1* | Forward | 5′-AAATCATCCGACTCCTCGTC-3′ |
|  | Reverse | 5′-ggctccaacaaggtaattcg-3′ |
| *HPRT* | Forward | 5′-GGAGCGGTAGCACCTCCT-3′ |
|  | Reverse | 5′-CTGGTTCATCATCGCTAATCAC-3′ |
